# Machine Learning for the Dynamic Positioning of UAVs for Extended Connectivity

**DOI:** 10.3390/s21134618

**Published:** 2021-07-05

**Authors:** Francisco Oliveira, Miguel Luís, Susana Sargento

**Affiliations:** 1 Department of Electronics, Telecommunications and Informatics (DETI), University of Aveiro, 3810-193 Aveiro, Portugal; fbpo@ua.pt (F.O.); susana@ua.pt (S.S.); 2 Instituto de Telecomunicações, 3810-193 Aveiro, Portugal; 3 ISEL-Instituto Superior de Engenharia de Lisboa, Instituto Politécnico de Lisboa, 1959-007 Lisboa, Portugal

**Keywords:** unmanned aerial vehicle, UAV positioning, machine learning, wireless communications

## Abstract

Unmanned Aerial Vehicle (UAV) networks are an emerging technology, useful not only for the military, but also for public and civil purposes. Their versatility provides advantages in situations where an existing network cannot support all requirements of its users, either because of an exceptionally big number of users, or because of the failure of one or more ground base stations. Networks of UAVs can reinforce these cellular networks where needed, redirecting the traffic to available ground stations. Using machine learning algorithms to predict overloaded traffic areas, we propose a UAV positioning algorithm responsible for determining suitable positions for the UAVs, with the objective of a more balanced redistribution of traffic, to avoid saturated base stations and decrease the number of users without a connection. The tests performed with real data of user connections through base stations show that, in less restrictive network conditions, the algorithm to dynamically place the UAVs performs significantly better than in more restrictive conditions, reducing significantly the number of users without a connection. We also conclude that the accuracy of the prediction is a very important factor, not only in the reduction of users without a connection, but also on the number of UAVs deployed.

## 1. Introduction

Unmanned Aerial Vehicle (UAV) networks are receiving increasing attention mostly due to their potential in a number of innovative services and applications, which are useful not only for military, but also for public and civil purposes. They are useful, mainly due to their versatility and dynamic nature, as their nodes and links can change, along with their positions, according to their needs. For this reason, they are a good candidate to reinforce existing networks when they fail or are overloaded [1].

The beauty of UAV networks lies in the fact that the position of the network nodes, the UAVs, can be controlled, which can be dynamically changed to optimize the network performance, and according to the users’ needs and their mobility. To find the best placement for the UAVs, the UAV network should be able to extend the base network with the limited capacity, by either moving the UAVs according to the needs, in real-time, or predicting ahead of time where the users will be and how they will move. The first method has the advantage of allocating exactly what is needed for the current situation, but has the disadvantage of the delays associated with UAVs positioning. The second method does not have any delays, as it can mobilize the UAVs ahead of time and optimize their position according to predicted movements; however, the accuracy of the prediction will influence how well the UAV network can help the existing network. Moreover, the volatile nature of the UAVs network, and the fact that UAVs are energy-restricted nodes, require that the placement needs to be determined with a low-complexity approach and be adapted over time.

Considering the previous requirements, this paper proposes an algorithm to calculate the best positions for UAVs in the network, in order to provide a connection to as many users as possible, satisfying their services and requirements. The algorithm predicts, through machine learning approaches the users’ position, and according to their needs, it predicts the position of the UAVs through machine learning, according to the predicted requirements and location.

Several machine learning approaches are tested to determine the users’ positions. Random Forest and Gradient Boosting have the best results, while Lasso, Ridge and ElasticNet are tied at the last place. In terms of the time to train and predict, which is a very important metric, Random Forest has a training time almost five times higher and a prediction time 36 times higher, than Gradient Boosting.

The proposed algorithm for UAVs positioning is tested with real data of the users’ location and their connection through base stations. The test comprises the influence of several parameters, such as the base station bandwidth and UAVs network range, to understand the optimal conditions for the use of a UAV network to extend an already existing network of ground stations, and in which situations should the algorithm be used. The results show that the algorithm performs better in less restrictive network conditions, and that the accuracy of the prediction is indeed an important factor to improve the users connectivity.

The main contributions of this paper are summarized as in the following:

- Test a set of machine learning approaches for user mobility prediction with real sets of users and their location;
- Proposal of a UAV positioning algorithm able to identify overloaded areas and areas that can receive more user’s traffic, considering the users’ positioning, users’ mobility and each users’ needs;
- Build of a bridge of UAVs that is able to transport the traffic through underloaded areas, in a dynamic approach and updated over time;
- Test the approach with the real set of users and with new metrics to understand the usefulness of UAVs to help in the transport of the users’ traffic.

The rest of this paper is organized as follows. Section 2 presents the related work. Section 3 describes the problem and our proposed approach. Section 4 explains the methodology for choosing the most suitable machine learning algorithm for the users’ prediction. Section 5 describes the proposed algorithm, while the results are presented in Section 6. Finally, Section 7 concludes this paper and presents the future work.

## 2. Related Work

In UAV networks, the coverage optimization is always a challenge that needs to be addressed. The network coverage optimization problem has been confronted by a number of different research works, resulting in multiple possible solutions. Galkin et al. [2] investigated how different UAV network parameters, such as density and height above ground, as well as environmental parameters, such as the building density and building heights, can influence the coverage probability. Kuhlman et al. [3] proposed an automated, physics-aware planner that made use of an information value map for path planning with a Markov Decision Process-based approach. Lyu et al. [4], in a scenario with no ground base stations, proposed a solution where UAV-mounted mobile base stations provide wireless connectivity to a number of ground terminals. The algorithm places a priority on ground terminals on the boundary, to maximize coverage efficiency. Reina et al. [5] presented a solution based on a multi-sub-population genetic algorithm and compares it to single-population genetic algorithms and other meta-heuristic optimization algorithms, and found that the multi-sub-population genetic algorithm achieves better performance than the other options. Sabino et al. [6] also proposed a genetic algorithm, in this case, a Multi-Objective Evolutionary Algorithm, to optimize the UAV node placement considering minimization of the number of UAVs, assuring the necessary bandwidth and a limited number of UAVs. Khan et al. [7] proposed a system where coverage of an area is given to a UAV that can handle the specific quality of service requirements. Each UAV’s capability to provide the required quality of service is evaluated through a reputation-based auction mechanism. They also address the real-time monitoring framework challenge present in this solution by using a permission blockchain architecture considering Support Vector Machine.

Other works also take into account the energy management of the UAVs as an important component to the network coverage optimization. Peng et al. [8] proposed an algorithm based on an Echo State Network to predict trajectories of user equipments, and a Kuhn–Munkres-based algorithm to find the most energy-efficient trajectories for the UAVs. Liu et al. [9] used Deep Reinforcement Learning for both the control of the UAVs and for controlling energy consumption, communications coverage and connectivity. The work in [10] proposed a framework to derive the localization error of terrestrial nodes in urban areas when using UAVs as anchor nodes. Such framework includes height-dependent UAV to ground channel characteristics and a highly detailed UAV energy consumption model. Following a different problem in [11] the authors derive a model to minimize the total UAV energy consumption while satisfying the communication throughput requirement of each ground user. The model includes both propulsion energy and communication-related energy. The work in [12] presents a detailed survey on the applicability of ML techniques for UAV-based communications.

However, all these works consider a static network of UAVs. In a real network, users are moving, and the needs of UAVs also move with the users. This paper proposes an approach to predict this movement, and move the UAVs in advance to their best locations.

## 3. Problem Statement

In a network, traffic is not uniformly distributed across all base stations, with people naturally migrating more to some areas and less to others. This will cause some base stations to have more users than they can handle, while other base stations are largely unused, causing connection problems to some users, even though the network as a whole has more than enough resources to accommodate all users.

**Figure 1:**
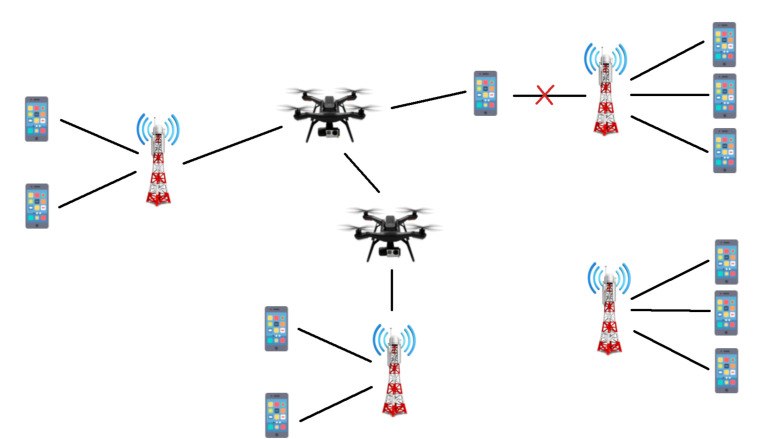
UAV assisted communication network.

By identifying where the network will be overloaded, we will redistribute the traffic of overloaded areas to places that have a lesser strain on the network, in an attempt to make the distribution of users throughout the city as uniform as needed. This is the purpose of the UAVs in the network: to divert the traffic to underloaded areas, so that all users can be connected with their services and requirements. The UAVs will then be placed in the best areas to redistribute the traffic in the network, as illustrated in Figure 1.

To identify the overloaded areas in the network preemptively, we will use a machine learning-based prediction algorithm. Several machine learning techniques are tested, and the one with the best performance will be chosen. To be able to train it, we need a suitable dataset that has records of a variety of users with information about the position, connection type and quality, with fairly frequent updates, like once every 15 min. Furthermore, the data should also have detailed information about the positioning of the ground base stations, which service they provide, their capacity and the area they can provide connection to. Finally, this data should contemplate a location like a big city, with 50 to 100 km^2^.

## 4. Position Prediction Approach

The real data used in the user position prediction results from a large-scale research initiative called Mobile Data Challenge (MDC) [13,14]. The dataset was collected with the Lausanne Data Collection Campaign (LDCC), which was responsible for providing close to 200 volunteers with data collection software on their smartphones. The data collection started in October 2009 and finished in March 2011.

Using this dataset, we extracted the information necessary for training, which consisted of the position of the users over time. We focused all the data on a circular area in the city of Lausanne with a 5 km radius. This area was then divided into rectangles of approximately 200 m horizontally and 140 m vertically.We also divided the data according to its date: month, weekday and hour.

**Figure 2:**
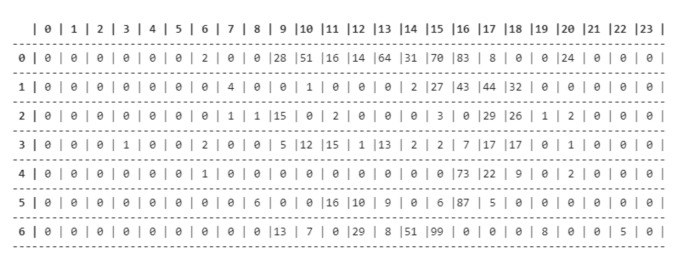
Example of the number of users in the matrix format.

The end result is a four-dimensional matrix in which the first dimension comprises the coordinates that represent the rectangular section, the second dimension is the month, the third is the weekday and the fourth is the hour, each cell containing a number that represents the quantity of users. Figure 2 shows an example of the matrix for an area in May, where each line represents a different weekday, by the numbers 0 to 6, and each column represents a different hour, from 0 h to 23 h.

### 4.1. Feature Selection

To perform feature selection, we use a method called Boruta [15], which compares the features to a randomized version of themselves, called shadow feature, only deeming them as useful if the features can perform better than their shadows. Performing better than the shadow becomes a very definite threshold for this method, that does not need any human input. Besides that, the algorithm also runs several times, to understand how each feature performs favorably and how many times it does not. Since the result for each run is a “useful” or “not useful” criterion, the results follow a binomial distribution and the features are positioned on it. Boruta then divides the binomial distribution into three different areas which are the features to drop, features to keep tentatively and features to keep. The features to drop and features to keep areas are defined by being the tails of the distribution (the extreme portions of the distribution), where each tail represents 0.5% of the distribution. Features in the features to drop area should be dropped as they have little value as predictors. Features to keep should be kept, as they are very useful for the prediction. The features to keep tentatively are the features that the Boruta method could not guarantee that they are useful, and should be used according to the discretion of the person building the model. In the case of this dissertation, those features will be kept, since the number of features is not very high.

The Boruta algorithm is executed using a random forest regressor as the estimator, used to evaluate if a shadow feature is better or not than the feature, for 20 iterations. The results can be seen in Table 1.

Features in the keep tentatively category are not guaranteed to be useful, and they are left for human responsibility. In this case, the feature “Weekday” was kept.

### 4.2. Machine Learning Algorithms and Hyper-Parameters

The machine learning methods used in the users’ mobility prediction are shown in Table 2. They are separated into three different categories, which are Linear, Ensemble and Deep Neural Network. Ensemble learners use simpler machine learning algorithms, like the decision trees in this case, and improve them either by averaging the values of several algorithms, or by continuously adjusting bias values on the course of several iterations of the same algorithm.

For all the machine learning algorithms except neural networks, the sklearn python library is used. This library provides implementations of the algorithms with default values on most algorithms that were not changed, unless it is specifically mentioned in the remaining manuscript.

#### 4.2.1. Lasso, Ridge and Elastic Net

The sklearn python library offers implementations of these three algorithms with built-in cross-validation. This means that the algorithm uses the cross-validation capabilities to select the best hyper-parameters, in order to build the best model according to the available data. therefore, the choice of the hyper-parameters was left entirely to the responsibility of the algorithm.

#### 4.2.2. Random Forest Regressor

**Figure 3:**
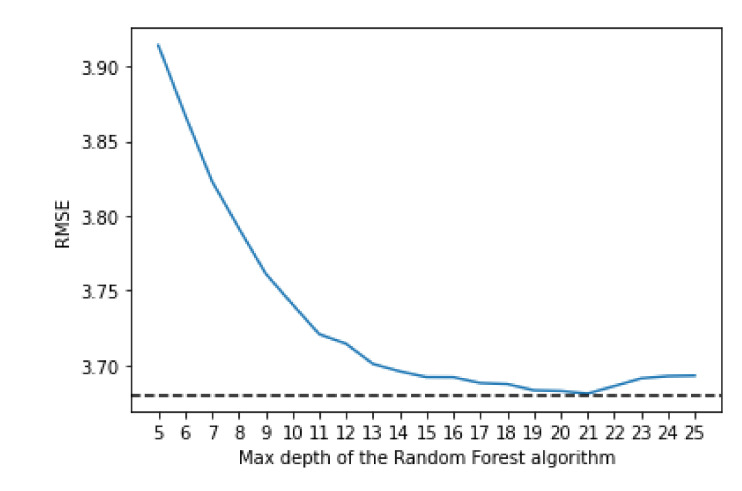
Comparing Root-Mean-Square Error (RMSE) values with different values of max depth for the Random Forest algorithm.

**Figure 4:**
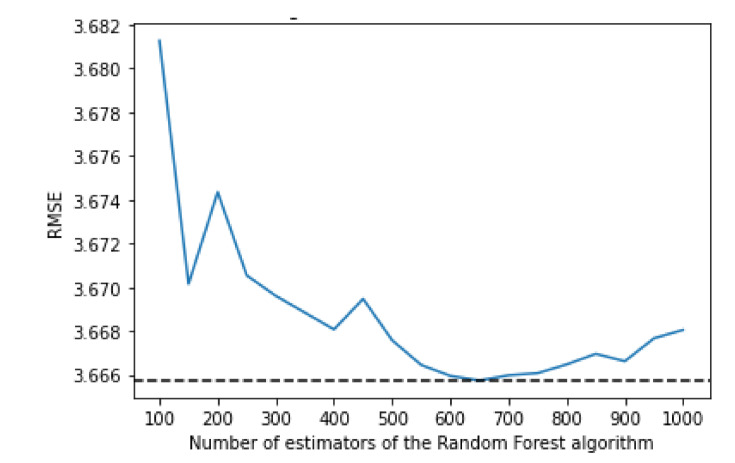
Comparing RMSE values with different values of estimators for the Random Forest algorithm.

The random forest uses a number of decision trees with various sub-samples of the dataset; it then uses the average of them all to improve the predictive accuracy and to control over-fitting. To decide on the value for the maximum depth and the number of estimators, an empirical research was done. The algorithm was trained using various values of both max depth and number of estimators which can be seen in Figure 3 and Figure 4, respectively.

For the max depth, the optimal value seems to be 21, even though the differences in the performance start to be very small after 15. It is worthwhile to note that a higher depth also implies a bigger training time; therefore, if a small training time is needed, a smaller value of depth, like 15, might also be acceptable, as the difference in performance does not make a significant impact.

Considering the number of estimators, the results are more irregular, but the number which offers the best performance is 650, at the cost of a significantly bigger training time when compared to, for example, using 150 estimators (4 h versus 52 min).

#### 4.2.3. Gradient Boosting Regressor

Gradient boosting is an ensemble machine learning technique that uses a number of weaker prediction models to build a stronger prediction model. For each model, it tries to find a new estimator that would reduce the error of each prediction. This estimator is adjusted iteratively, going through each prediction model. The final result is a single, stronger, prediction model, that predicts with higher accuracy than any of the weaker prediction models that it uses. Like the Random Forest algorithm, some parameters will also be tested empirically, to research their impact on the performance of the model.

**Figure 5:**
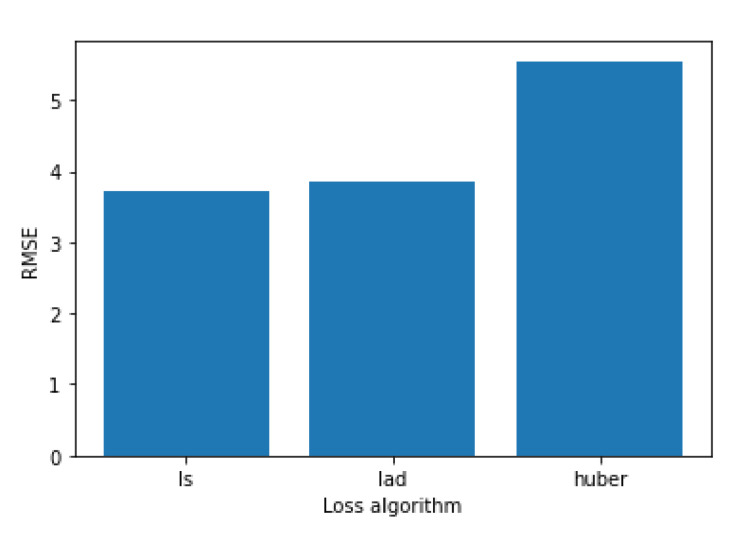
Comparing RMSE values with different loss algorithms for the Gradient Boosting algorithm.

**Figure 6:**
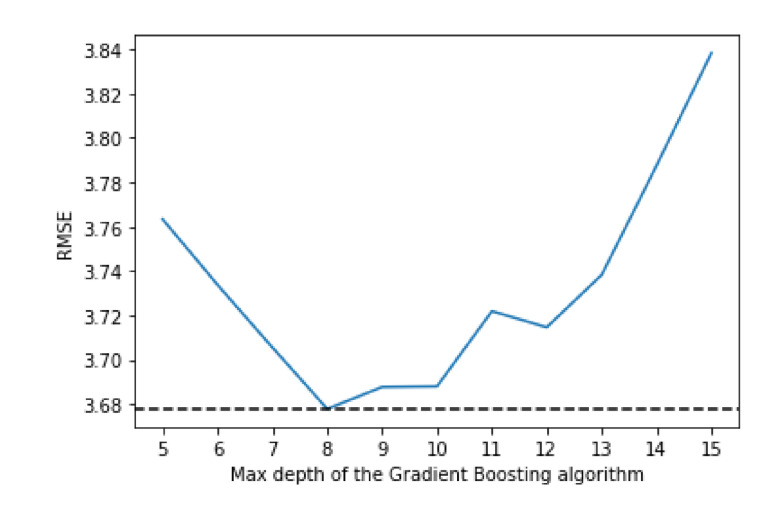
Comparing RMSE values with different values of max depth for the Gradient Boosting algorithm.

**Figure 7:**
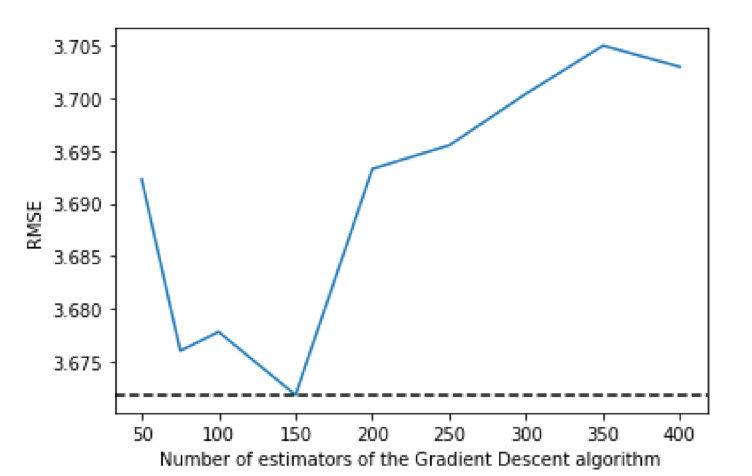
Comparing RMSE values with different values of estimators for the Gradient Boosting algorithm.

Figure 5 shows how the three available loss algorithms perform, where “ls” stands for least squares regression, “lad” stands for least absolute deviation and “huber” is a combination of the two. While the “huber” function performs notably worse, “lad” and “ls” have more similar performances, but “ls” achieve better performance. Figure 6 shows that the best value for the max depth is 8, while Figure 7 shows that 150 is the number of estimators that have the best performance.

#### 4.2.4. Neural Networks

The neural network uses nodes, which is where the computation happens, divided into layers. Besides the input layer and the output layer, a neural network may have several layers, each one with several nodes. To decide both the number of layers and the number of nodes in each layer, several different models were tried and evaluated.

**Figure 8:**
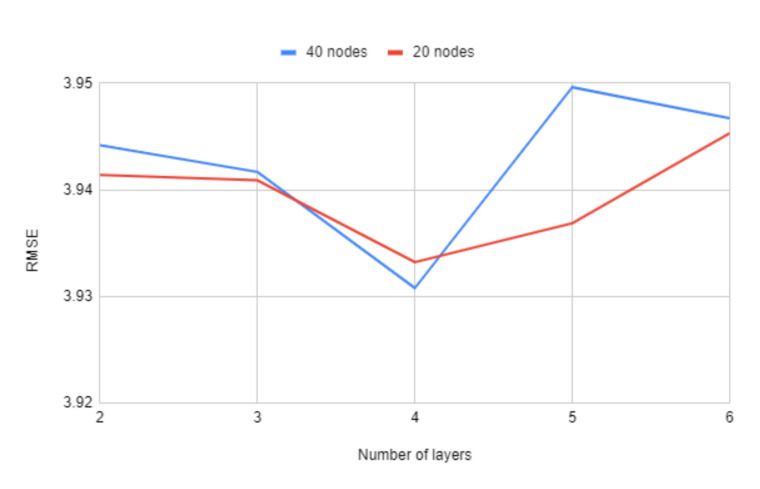
Comparing RMSE values with different number of layers and nodes per layer for the neural network.

In Figure 8 we can observe the impact that the number of layers has on the RMSE values. While it does not have a large variation, using four layers seems to consistently offer the best results.

**Figure 9:**
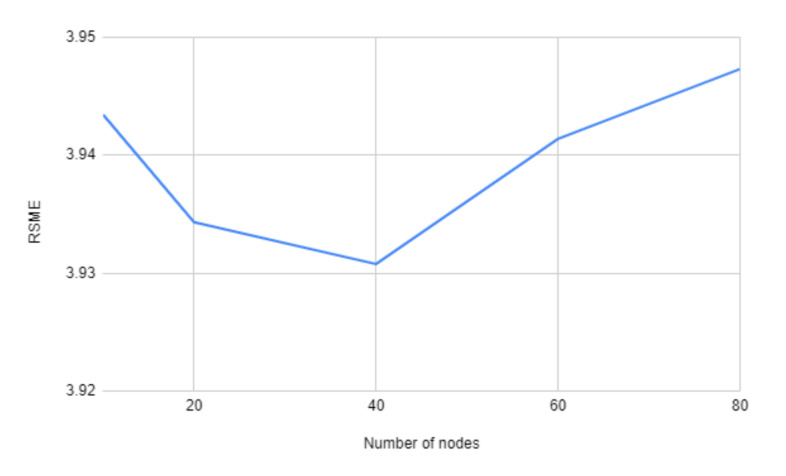
Comparing RMSE values with different number of nodes per layer for the neural network.

In Figure 9 we use four layers for each model, but varying the number of nodes from 10 to 80. Here in this figure, the variation is also very small, but using 40 nodes gives the best RMSE score.

**Figure 10:**
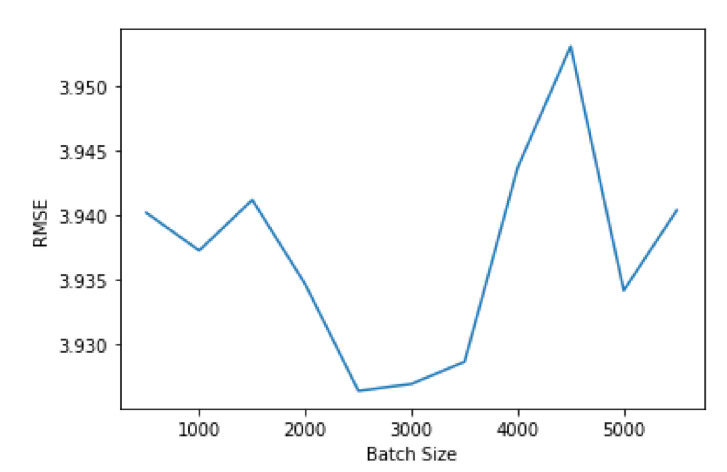
Comparing RMSE values with different number of batch size for the neural network.

Another aspect that impacts the performance of a neural network is the batch size. Figure 10 shows the variation in performance according to the batch size. The variation is highly irregular, not converging to any value, as shown in Figure 10. Since it is not practical to test every possible value for the batch size, the best option is to try to find a local minimum that offers good performance when compared to the other local minima. Therefore, a batch size of 2500 offers the best performance value.

With these results, we can come to the conclusion that the best neural network model for this data is one with four layers, with 40 nodes each, and with a batch size of 2500. This model is the one used in the performance tests in the following section.

### 4.3. Performance of the Machine Learning Algorithms

To compare the performance of the machine learning algorithms, we use metrics such as Mean Absolute Error (MAE), Root-Mean-Square Error (RMSE), R-squared (R^2^) as well as training and prediction times. We used 80% of the dataset for training, and the remaining 20% for testing, i.e., 13 months for training and 3 months for validation, with the data being shuffled previously.

**Figure 11:**
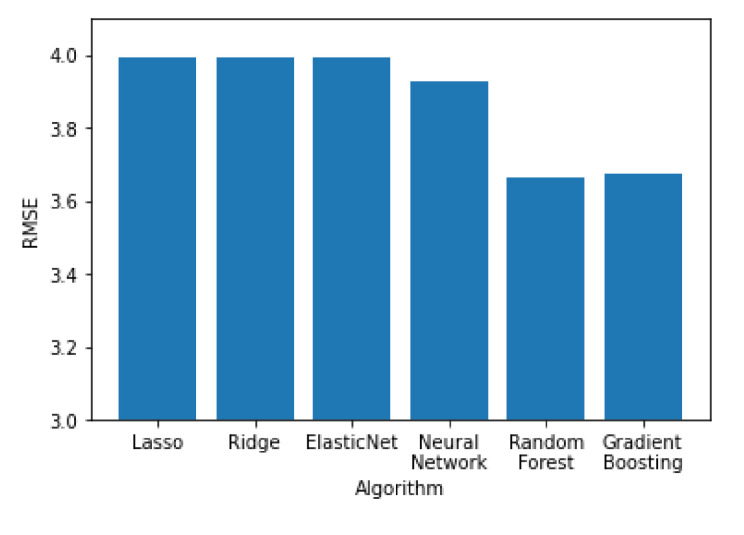
Performance of the algorithms according to their RMSE value.

**Figure 12:**
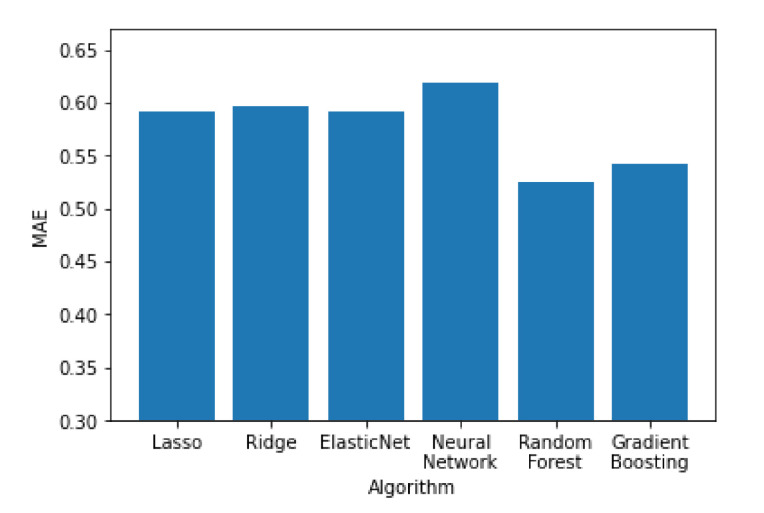
Performance of the algorithms according to their MAE value.

**Figure 13:**
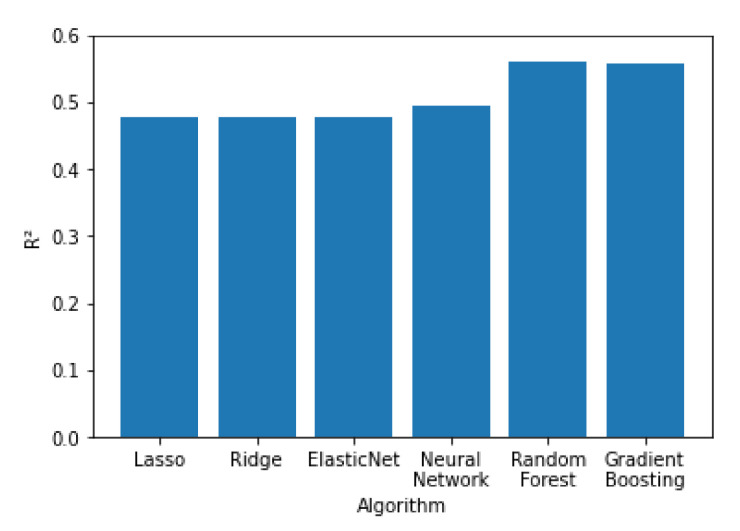
Performance of the algorithms according to their R^2^ value.

In Figure 11, Figure 12 and Figure 13 all the algorithms are compared according to their performance with the RMSE, R^2^ and MAE metrics, respectively. Random forest and Gradient Boosting have the best results, while Lasso, Ridge and ElasticNet are tied at the last place. All algorithms have RMSE values significantly higher than the MAE values, indicating a high variation on the error values with a significant portion of them being higher than the average. Random forest and Gradient Boosting have the best results, while Lasso, Ridge and ElasticNet are tied at the last place. All algorithms have RMSE values significantly higher than the MAE values, indicating a high variation on the error values with a significant portion of them being higher than the average.

Another important metric is the time required for training and predicting. Table 3 shows these values when training with approximately 5 million samples of data. Random Forest, that performs slightly above Gradient Boosting, has a training time almost 5 times higher and a prediction time 36 times higher. If prediction speed is a priority, then Gradient Boosting might be the best option, rather than Random Forest.

### 4.4. Prediction Results

**Figure 14:**
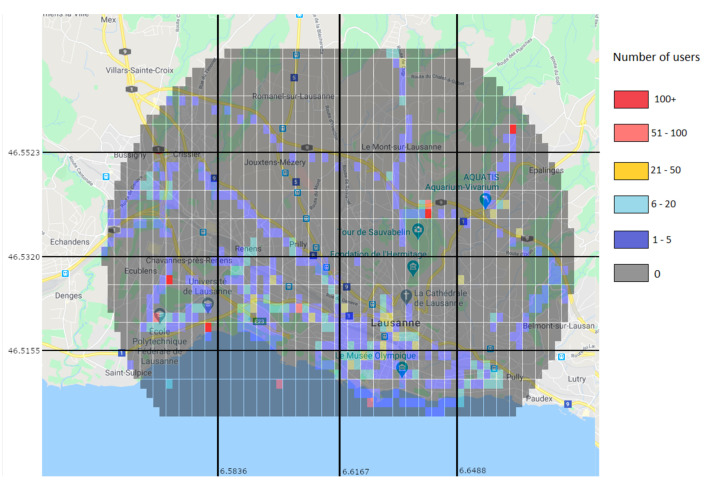
Real values of the number of users on an April Monday at 15–16 h.

**Figure 15:**
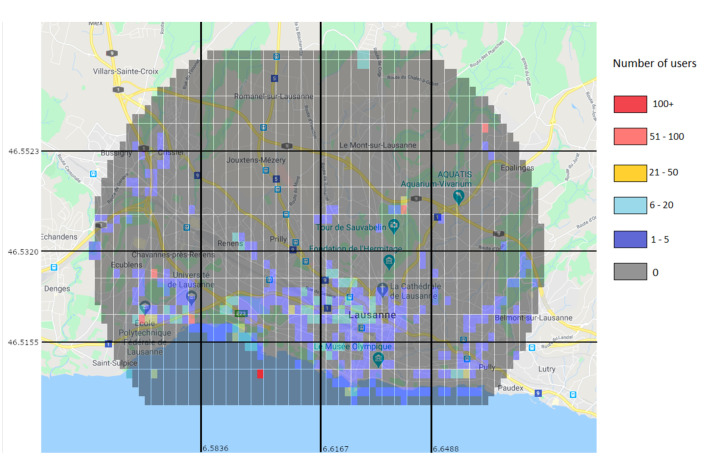
Prediction of the number of users on an April Monday at 15–16 h.

Through the prediction approach, the Random Forest predictor was used to generate a user distribution map. Figure 14 shows the real values for the 15–16 h interval, while Figure 15 shows the predicted values for the same interval. While the prediction shows user activity in the same regions than the real values do, it still misses some of the regions with low user values. For example, in Figure 14 there is a clear trace of users along a road in the top left region, which is not present in the predicted values. This is due to the low amount of users and data.

## 5. UAV Positioning for Network Traffic Redistribution

### 5.1. UAVs as Traffic Redistributors

Aerial UAVs are highly mobile and dynamic, which make them excellent to be used as traffic redistributors: they can follow the users as they move throughout the day. In order for them to redistribute traffic, they need to build a bridge of UAVs from the overloaded area to the target area. UAVs will not serve as base stations, but only as extensions of the already existing base stations. With this purpose, there will be UAVs with either one of these two functions: connecting to the users or providing interconnection between the target base stations and the UAVs responsible for connecting to the users. UAVs that connect to the users will be in the areas that are overloaded, moving with the users’ movement prediction, and will be the ones responsible for providing connection to those users. The UAVs that provide interconnection will transport the traffic towards the target base station in a less populated area. The number of UAVs will depend on the distance between the overloaded area and the target area, the range of the UAVs, the users’ needs and the links capacity according to the distance.

Each UAV is limited in its capabilities. The number of users each one can serve is limited by its bandwidth, transmission speed and services required by the users. While the bandwidth and transmission speed are fixed for each UAV, even though transmission speed might vary according to the number of interconnecting UAVs, the services required by the users will most likely differ from user to user. This variation will be taken into account when calculating the capacity of each UAV, and also the number of UAVs needed to satisfy the traffic needs of each area.

### 5.2. UAV Positioning Algorithm

The UAV positioning algorithm is responsible for identifying overloaded areas and areas that can receive more user’s traffic: through the mobility prediction of the users and the location of the base stations, decide to which areas traffic will be redirected and, therefore, decide the best placement of the UAVs.

The algorithm proposed in this paper has three main steps. After predicting the number of users in each area:

1. It identifies overloaded areas and iterates through all overloaded areas;
2. It finds the nearest areas to offload traffic; and
3. It builds a bridge of UAVs that transports the traffic to those areas.

#### 5.2.1. Identifying Overloaded Areas

To identify overloaded areas, the average user bandwidth requirements as well as the available bandwidth per base station are required to be known. With these two values, we can determine the maximum number of users per base station and use it to determine, for each base station, the number of users they can provide a connection to. Consequently, we can also calculate and predict, for each area, the number of users without access to enough bandwidth, if any. Algorithm 1 is proposed to run the previous steps.
**Algorithm 1:** Number of predicted users without a connection. **Output**: Number of users in excess in each area (extra_users_area) and the number of users connected to each base station (bs_users)

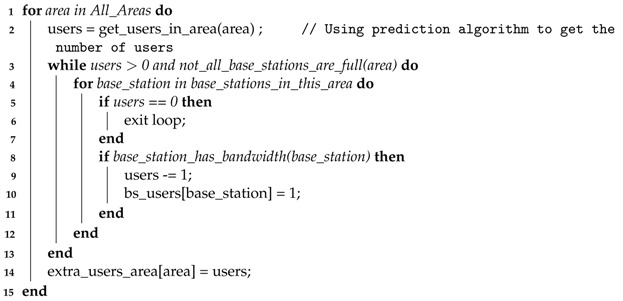



The algorithm assigns a base station to each user that is predicted to be inside it, until all base stations are saturated or until all users are distributed. Overloaded areas can now be predicted and used to distribute the UAVs.

#### 5.2.2. Finding the Nearest Areas to Offload Traffic

After identifying an area with predicted users in excess, we need to offload their traffic into other areas that can handle the traffic increase. For this purpose, we propose Algorithm 2 that finds a number of available areas closer to the overloaded area that requires the traffic offload. This number can be changed to define how many areas the algorithm will return to forward the traffic to, to reduce the probability of overloading any areas with this redistribution.
**Algorithm 2:** Areas available to receive more traffic. **Input**: Overloaded areas and amount of extra users per area
 **Output**: Areas available to receive offloaded traffic (list_of_available_areas)

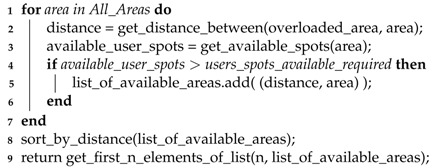



The loop iterates through all areas, from now on denominated as target areas, calculates the distance from the current area to the target area, and checks if it can receive the necessary traffic. If it can, then we add a new tuple to the list created, containing the distance calculated before and the identifier of the target area. When the loop finishes, we sort the list by the distance in order to select only the *n* closest target areas, and return them in a list format. Since each area may have multiple base stations covering them and two different areas may be covered by the same base station, it is possible that the areas returned will not be able to support all the traffic required, which will require preventive measures when using the output of Algorithm 2.

With this list, we can then proceed to redistribute the users through the areas, with the help of the aerial UAVs.

#### 5.2.3. UAVs Distribution

To redistribute traffic, UAVs are used as a path to other areas. The number of UAVs used should be minimized when possible, which requires the use of UAVs that were already placed before to serve as or complement paths to new areas. To avoid overloading any UAVs, each UAV has a counter on how many users it supports (considering the users’ requirements), so if it is needed, a reinforcement UAV is deployed. For this purpose, for every UAV that has been placed, there is a list of all other UAVs with a connection, not only directly, but also indirectly through other UAVs. Therefore, whenever Algorithm 2 returns a new area aiming to redirect the traffic, first it always checks if an available UAV path to the area already exists. If that is not the case, then we search for the shortest available path to find a UAV that has a connection closest to the target area. When this UAV is found, the amount of UAVs needed to reach the target area is then calculated according to the distance to the target area and the range of each UAV. UAVs are then placed in a straight line in intervals of length equivalent to their range. Finally, a list is created, which registers the position of every UAV, and the UAVs connected to.

#### 5.2.4. Uniting All Parts

With all key parts defined before, the entire system will now be integrated. In this integration, it is important to assure that the number of users connected to each base station will not surpass the ones that it can receive. The procedure to perform the integration and place the required UAVs to cover all predicted users in the network is described in Algorithm 3.

The algorithm contains a loop through all areas to verify if there are any users left without a connection, and if there are attempts to distribute them through other available areas. The list with the target areas is found using Algorithm 2, and then the algorithm iterates through all target areas, to check if a UAV route is already available, i.e., a path made of UAVs previously built. If this is not the case, the algorithm finds the UAV in the network closest to the target area (line 9) and, from it, it builds a path of UAVs to the target area. After a UAV path is established, the algorithm begins to distribute the user’s traffic through the base stations in the target area (line 15 to 26), and ends when all user’s traffic has been redirected to that target area, or if all base stations are already at their maximum capacity (considering each user’s traffic).

**Figure 16:**
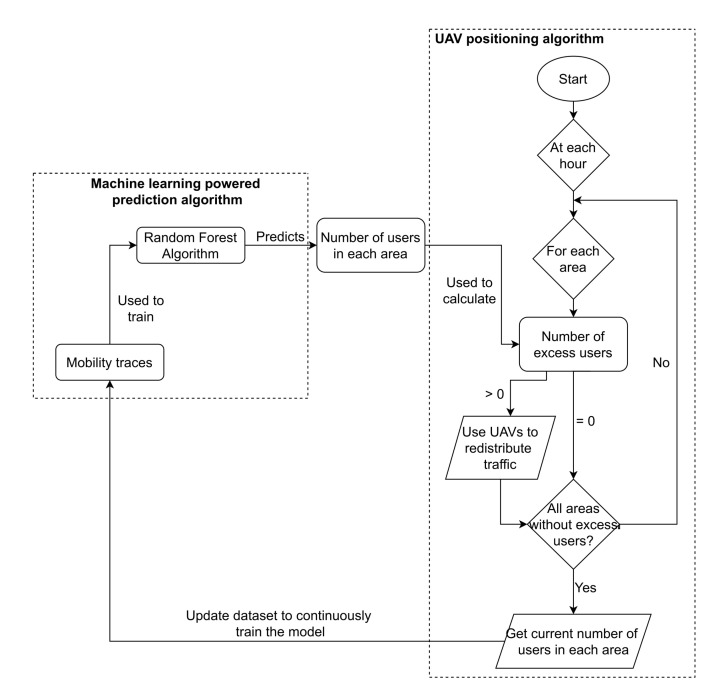
Overall workflow of UAV positioning system.

After the loop through all target areas, the distribution may not be able to provide connection to all predicted users in the current area, caused by two or more target areas sharing base stations, therefore providing less bandwidth availability than initially assumed. When this occurs, the algorithm performs the process again for the current area, finding other target areas and distributing the traffic through them, until there are no users left without a connection or all base stations are at their maximum capacity. The overall workflow of the proposed system, which encompasses all the algorithms presented before, is illustrated in Figure 16.
**Algorithm 3:** Location of the UAVs for traffic redistribution. **Input**: Number of users in excess in each area (extra_users_area) and predicted number of users per base station (bs_users)
 **Output**: UAVs distributed across the areas

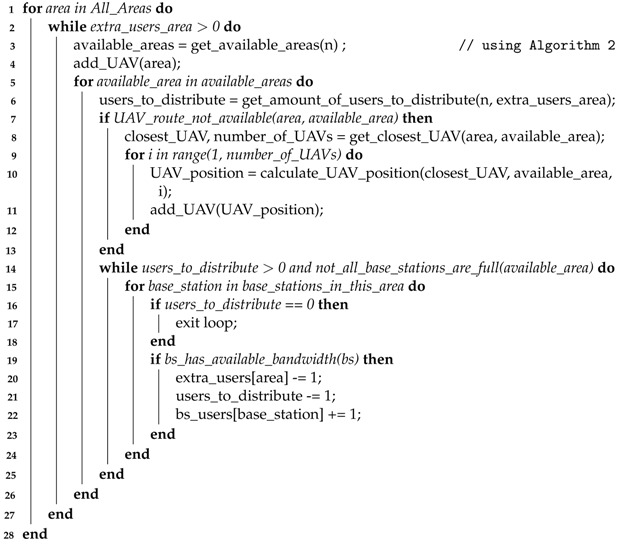



Finally, it is important to mention all the signaling required to make such a system to work. First, and to have an up-to-date ML model, the system needs to be constantly collecting the number of users per cell. This information can be done using tracking systems or the user’s information that can be gathered by the cellular stations. Such information must be gathered in a central entity that will be responsible for the use of the selected ML model. For every prediction stage, the updated positions must be transmitted to the UAVs in order to cope with overcrowded cells, which may include the deployment of new UAVs, the removal of unnecessary UAVs or simply the re-positioning of the UAVs in use. Resuming, in a real-time environment we have a system where the user’s positioning is gathered at a central entity (just like the system that gathered the MDC dataset), which is then used to periodically train the model. In that case, the UAV placement algorithm will be determining the best positions of the UAVs with historic and current users’ location and mobility, and will update their positions upon new placement results.

## 6. Performance Assessment

This section evaluates the performance of the algorithms by redistributing traffic through those UAVs, and checking how many users are left without a connection. We will now consider the real position of the users to check if the UAVs are placed in the best positions, that is, if the users’ mobility and location prediction were performed with good performance. The redistribution takes into account the positions of the base stations and UAVs, and tries to redistribute the traffic according to the UAVs distribution as a result of the UAV positioning algorithm.

The first step is to distribute the users through the base stations. After this process, the number of users in excess and users connected to each base station are known, and therefore, we should distribute the users in excess to other available base stations using the UAVs placed before.

To evaluate the performance of the proposed approach, a new metric is created, henceforth called score. To calculate this score, we will use the number of UAVs as well as the number of users left without a connection. This process will be explained further in the following section.

### 6.1. Score Formula

The proposed score formula takes into consideration the number of UAVs used and how many users were left without a connection. To calculate the final score, we start with 0 points, which is the best possible score. Then, we will add the number of users in excess and UAVs. However, one user in excess does not necessarily mean one more point to the score, because we can give different weights to the extra users or the UAVs depending on what we want to prioritize. For example, if the priority is to use the least amount of UAVs, then each UAV may add two or three points to the total score while each extra user only adds half or one point. On the other hand, if the priority is that no user is left without a connection, then each extra user may add two or three points while each UAV only adds half or one.

The results will be shown with different weights for the extra users $\left(w_{u}\right)$ and number of UAVs $\left(w_{d}\right)$, in order to reach the best conclusion regardless of the priority. The different score formulas are the following:

- Balanced—both inputs have the same weight, serving as a good baseline when there is not any special focus on either reducing the number of users without a connection or the number of UAVs to be placed $\left(w_{u}=w_{d}\right)$;
- User focused—a bigger weight is given to the number of users without a connection, while a smaller weight is given to the number of UAVs $\left(w_{u}=4w_{d}\right)$;
- UAV focused—the bigger weight is used for the number of UAVs $\left(w_{u}=0.25w_{d}\right)$.

Thus, the score is given by

(1) $$Score=\frac{\left(w_{d}\cdot n_{d}+w_{u}\cdot n_{u}\right)}{\Delta_{w}},$$

where $n_{d}$ represents the number of UAVs used, $n_{u}$ the amount of users with coverage by the extra users, and $\Delta_{w}$ a normalization factor.

To reflect about the usefulness of the UAV positioning algorithm, the score is also calculated before applying the algorithm, where the number of UAVs will inevitably be 0, so only the number of extra users will be used for the calculation. We then calculate the difference between the beginning and the end of the redistribution, and measure the percentage by which the score changed. When a bigger weight has been given to the number of UAVs, the score may actually be higher after applying the algorithm, and the percentage will be negative.

### 6.2. Results

To better understand the strengths and weaknesses of the proposed approach, tests were performed changing the base station available bandwidth, the user bandwidth and the ranges of the UAVs. For each set of parameters, the score was calculated through the three strategies presented before.

The placement algorithm was executed five different times, corresponding to 5 h, from 14h to 19h, and the score results were averaged. The data used in the performance assessment was taken from the dataset used to train the prediction algorithm. The prediction is executed hourly, using the Random Forest method, as well as the UAV positioning algorithm, calculating the score for every hour, and averaging it in the end. It is assumed that the users require a fixed amount of bandwidth for the duration of the hour, but this value may change in the subsequent hour.

The bandwidth required for each user is assumed to be randomly selected with a given probability. For example, for the standard example of an average user bandwidth of 50, the possible values are 25, 50 and 75, all with a probability of 33.3%. The bandwidth has no explicit unit, but they all use the same unit and are therefore comparable to one another.

The results will show the score obtained or the percentage decrease when comparing the network before and after applying the algorithm. Each mark corresponds to the mean of 5 runs and its 95% confidence interval (the confidence intervals are small, and may not be visible in some of the points).

#### 6.2.1. Base Station Bandwidth

This sub-section evaluates the impact of the base station bandwidth. For this set of results, the average user bandwidth is 50 and all UAVs are considered to have a range of 240 m.

**Figure 17:**
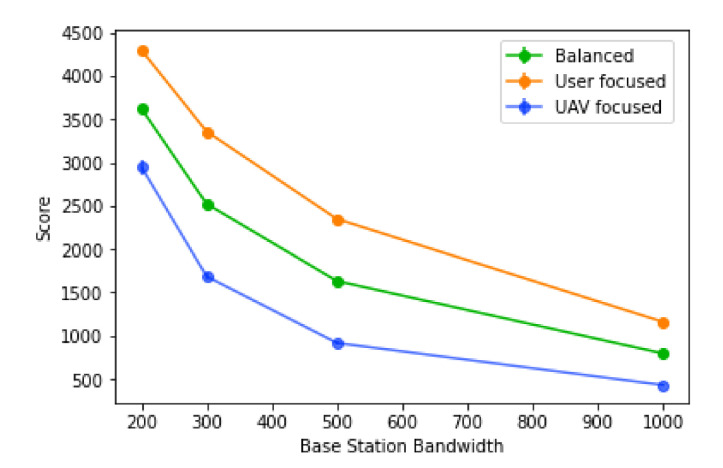
Score of the algorithm with different values of base station bandwidth.

The bandwidth available in each base station is a very strong factor in how high the score is, with the biggest score values coming from lower values of the base station bandwidth, as observed in Figure 17. This is due to a higher number of UAVs placed, since more user’s traffic will have to be redirected; it is also due to a lower tolerance for mistakes in the prediction, since there will be a lower number of base stations that can receive traffic from other areas.

**Figure 18:**
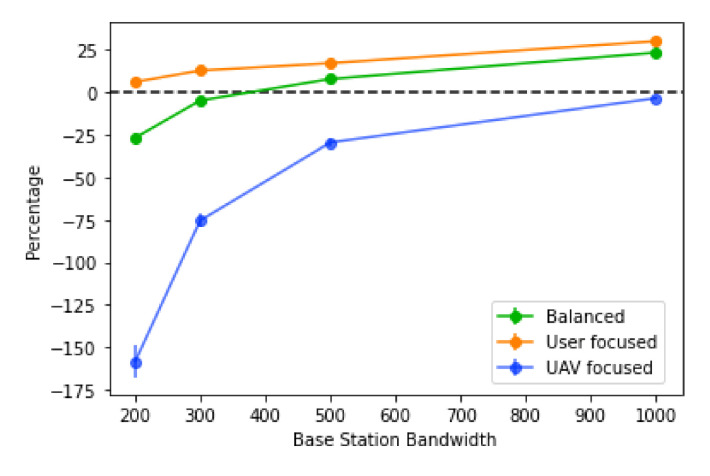
Percentage decrease of the score with different values of base station bandwidth.

The algorithm is also more efficient for larger values of base station bandwidth, with higher values also having a stronger percentage decrease in the score, as observed in Figure 18. However, for all types of score, the trend is the same: the actual values are very different depending on the score type. In the “user focused” type, the score always decreases, never going into negative values, where the same cannot be said for the “balanced” type, where the percentage goes negative towards the lowest values of the base station bandwidth. Finally, for the “UAV focused” type, the values tend to be worse, going into negative values when the base station bandwidth values reach 500 or less. The efficiency of the algorithm using this score is also lower overall, when compared to the other two. This suggests a sharp increase in the number of UAVs placed when the base station bandwidth decreases, caused by more users needing traffic redistribution and also more routes created, since each base station will also accept less traffic.

#### 6.2.2. Average User Bandwidth

On the user bandwidth evaluation, the bandwidth of the base station was set to 1000, and the average user bandwidth varied between 33 (each user with a value of 25 with 67 % probability, and 50 with 33% probability), 50 (with 25, 50 and 75 all with equal probability), and 75 (each user consuming either 50 with 33% probability or 75 with 67% probability). All UAVs are considered to have a range of 240 m.

**Figure 19:**
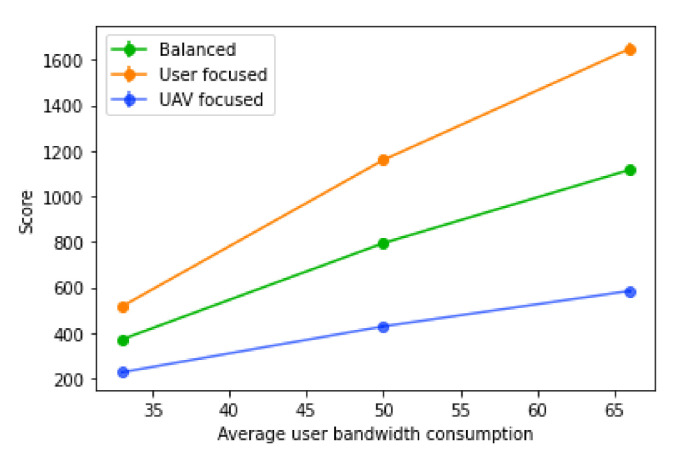
Score of the algorithm with different values of average user bandwidth.

The variation of the average user bandwidth has the opposite impact when compared to the base station bandwidth, as it just increases or decreases the number of users that fit into each base station. Naturally, as the average user bandwidth decreases, so does the score, since more users will be able to fit in the same number of base stations, as shown in Figure 19. With an increase in the average user bandwidth, less users will fit into each base station, increasing the number of users without a connection, and consequently, the number of UAVs placed. With higher values of average user bandwidth, less base stations will be available and less users will be hosted, therefore decreasing the tolerance for prediction errors.

**Figure 20:**
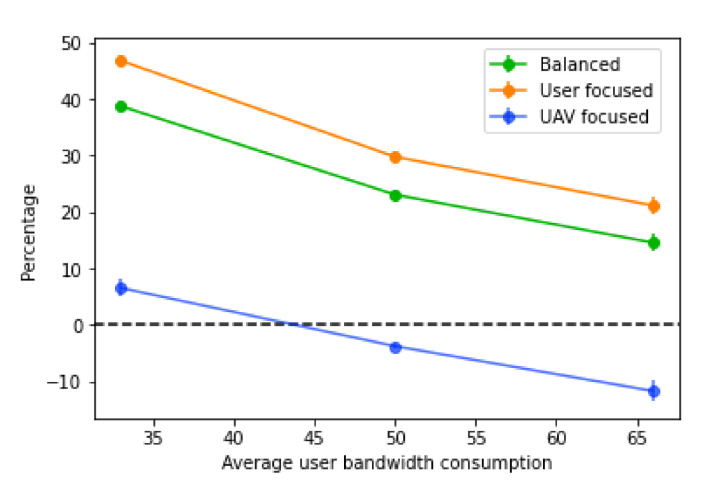
Percentage decrease of the score with different values of average user bandwidth.

Again, when comparing the decrease percentage (Figure 20), the results are very similar to what happens with the base station bandwidth variations. In this case, as the average user bandwidth consumption increases, the decrease percentage of the score gets lower. Moreover, it is noticeable that, with the “UAV focused” score, the percentage decrease is substantially lower than the other two types, which are very similar overall. This suggests that the main factor for the lower performance is the high number of UAVs placed, since with more users without a connection, and less availability on base stations to receive traffic, more UAV paths have to be built to redistribute the traffic.

#### 6.2.3. UAV Coverage Range

This test evaluates the influence of the UAV coverage range, where the range varied between 100, 240 and 500 m. The base station bandwidth was set to 1000 and the average user bandwidth is 50 with 25, 50 and 75 as possible values, all with equal probability.

**Figure 21:**
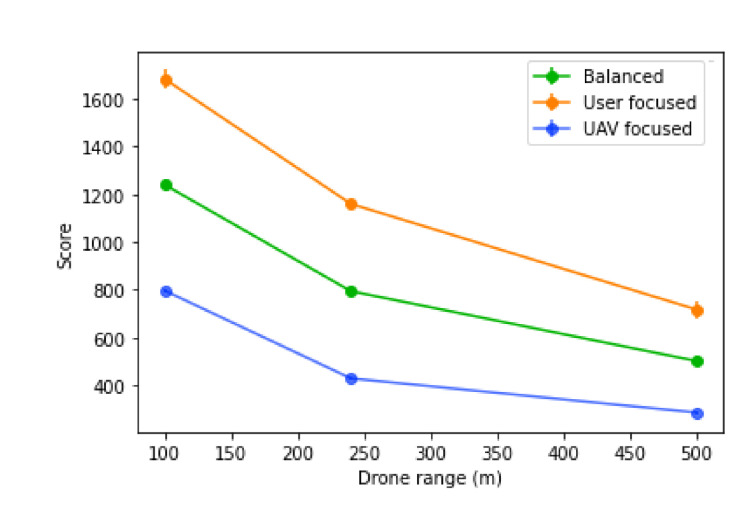
Score of the algorithm with different values of UAV coverage range.

The UAV coverage range will determine two key aspects: the number of UAVs needed to reach a remote base station, which will influence the number of UAVs needed in total; and also how tolerant to prediction errors the positioning of the UAVs is, which will influence the number of users left without a connection. With this in mind, we can expect that scores will decrease as the ranges increase, as shown in Figure 21.

**Figure 22:**
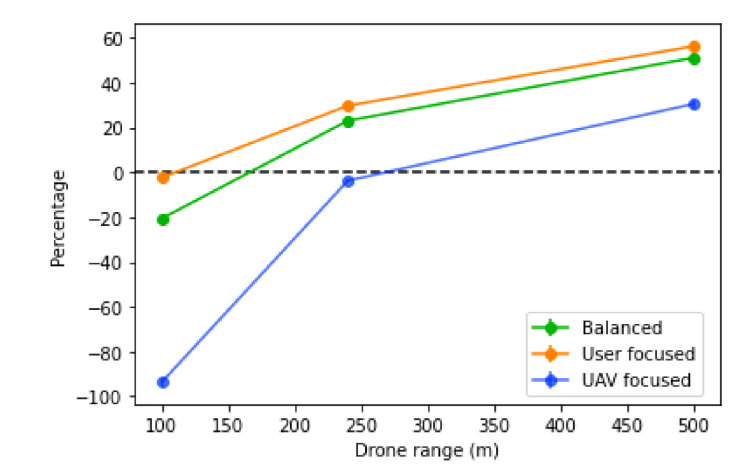
Percentage decrease of the score with different values of UAV coverage range with a base station bandwidth of 1000.

In the decreased percentage of the score, illustrated in Figure 22, similar results are observed. The decreased percentage of the score increases with the range. The UAV coverage range highly influences the performance, since the larger it is, the more tolerant the network is to bad UAV positioning originated through inaccurate prediction. While it does not change the amount of users’ traffic each base station can receive or the amount of traffic that exists, it changes how well the network can adapt to the current situation and user distribution, and also the number of UAVs placed.

#### 6.2.4. Predicted Users Vs Real Users

**Figure 23:**
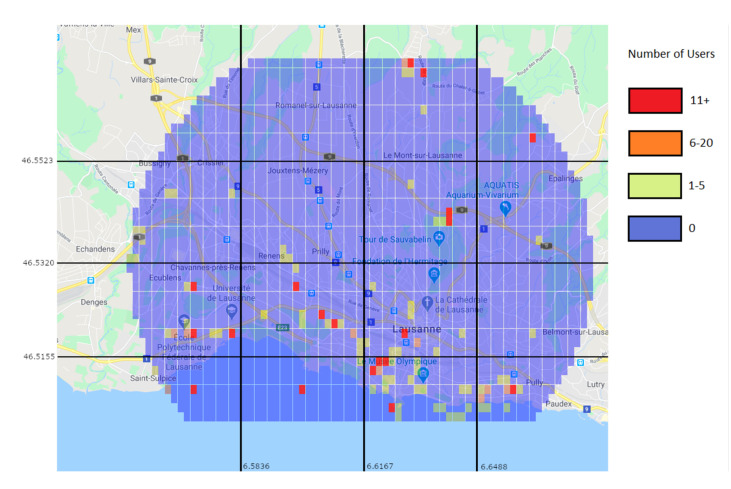
Prediction of users left without a connection (color marks).

**Figure 24:**
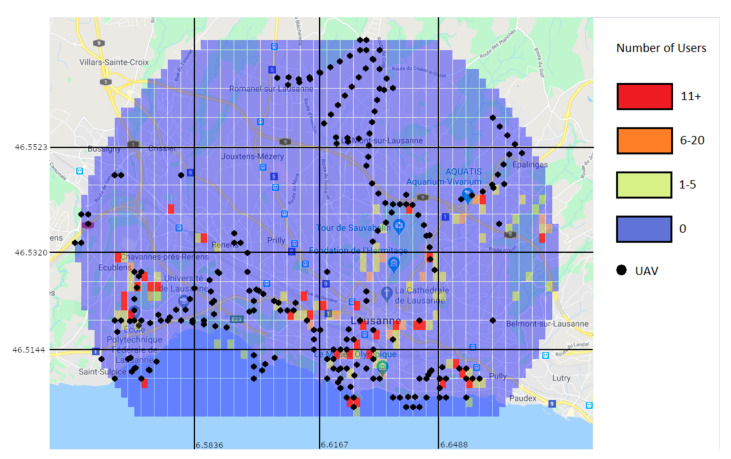
Users left without a connection (color marks) and positioning of the UAVs after the traffic redistribution (black marks).

To further illustrate the situation and the conditions in which the algorithm operates, Figure 23 and Figure 24 show the positions and number of users without a connection in various situations. Figure 23 shows them as the machine learning algorithm predicted, and Figure 24 shows the real number of users without a connection after the traffic distribution, and the positions of the UAVs. The most important aspect to take from the figures is that the prediction algorithm returns less users without a connection than in the reality, which will inevitably lead to a significant number of users without a connection after applying the UAV positioning algorithm.

As illustrated in Figure 24, there is still a significant number of areas that are not directly covered by a UAV, meaning that the prediction algorithm failed to correctly determine the number of extra users in those areas. While this can be improved by the use of a dataset with a large number of users, there are also other options that can lessen the impact of a less accurate prediction. One option is to reduce the threshold for the number of users needed until an area can be considered overloaded. Doing this will increase the number of UAVs placed, but also reduce the impact the prediction algorithm will have when it underestimates the number of users in an overloaded area. Another option is to allow UAVs to make some moving decisions after they are placed. This means that UAVs will be able to move to neighbouring areas if the movement would be beneficial overall.

**Figure 25:**
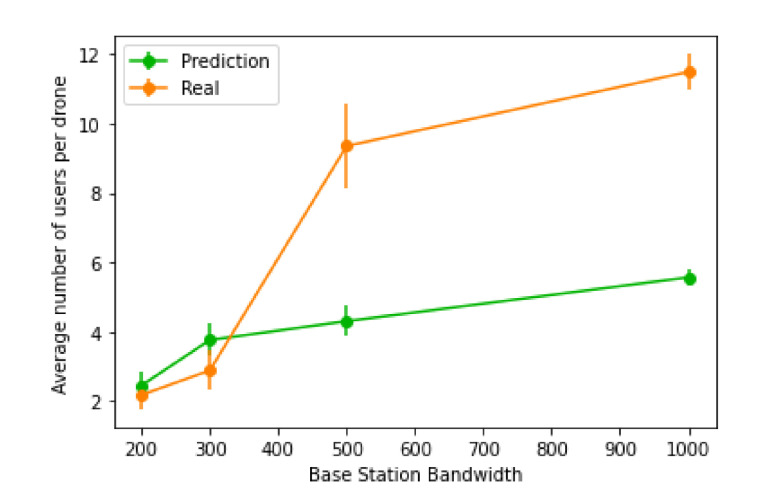
Average number of users per UAV when using real values versus predicted values.

Furthermore, we also investigated how many users each UAV was connected to, on average, which is illustrated in Figure 25. Using real values will generally achieve a much larger number of users per UAV, since the amount of users that benefit from the UAV placement will be significantly larger than the extra amount of UAVs placed. However, the same does not apply for more restrictive network parameters, as the number of extra UAVs placed starts to be higher than the amount of users it benefits, causing a decline in the number of users per UAV.

## 7. Conclusions

This paper presented an approach to predictively place and move aerial UAVs in a communication network to extend and balance the users’ traffic. It investigated the performance of multiple machine learning algorithms to predict the users’ location and movement. The performance results showed that the random forest and gradient boosting presented the best performance, with random forest having a bigger prediction and training time. Moreover, this paper designed and developed a UAV positioning algorithm that returns the positions of the UAVs in a UAV network, with the objective of extending an existing network and balancing the traffic load. This algorithm was tested with real and predicted data. We concluded that the prediction algorithm slightly underestimates the number of users in most areas, causing the UAV positioning algorithm to position less UAVs than it should, and in turn increasing the amount of users left without a connection. The UAV positioning algorithm performs favorably for less restrictive network conditions.

Future work will focus on using the prediction algorithm on areas with larger number of users, and study the impact that the signal propagation, interference, noise, fading and other impairments of the wireless medium have on the UAV network created by the UAV positioning algorithm. We will also consider the UAV energy and power consumption in the UAV placement algorithm, as well as the cost of its replacement.

## Figures and Tables

**Table 1:** Boruta results.

Feature	Decision
Month	Drop
Weekday	Keep tentatively
Hour of the day	Keep
Latitude	Keep
Longitude	Keep
Last known value	Keep

**Table 2:** Machine learning in the users’ mobility prediction.

Method	Type
Lasso	Linear
Ridge	Linear
Elastic Net	Linear
Random Forest	Ensemble
Gradient Boosting	Ensemble
Neural Network	Deep Neural Network

**Table 3:** Training and prediction times for all tested algorithms.

Algorithm	Training Time (s)	Prediction Time (s)
Random Forest	14,242	179
Gradient Boosting	2842	5
Neural Network	323	23
Lasso	28	0.02
Ridge	4	0.02
ElasticNet	27	0.02

## Data Availability

Restrictions apply to the availability of these data. Data was obtained from Idiap Research Institute and are available at https://www.idiap.ch/en/dataset/mdc. with the permission of Idiap Research Institute.
